# Light Suppresses Sporulation and Epidemics of *Peronospora belbahrii*

**DOI:** 10.1371/journal.pone.0081282

**Published:** 2013-11-27

**Authors:** Yigal Cohen, Moshe Vaknin, Yariv Ben-Naim, Avia E. Rubin

**Affiliations:** The Mina and Everard Faculty of Life Sciences, Bar-Ilan University, Ramat-Gan, Israel; Agriculture and Agri-Food Canada, Canada

## Abstract

*Peronospora belbahrii* is a biotrophic oomycete attacking sweet basil. It propagates asexually by producing spores on dichotomously branched sporophores emerging from leaf stomata. Sporulation occurs when infected plants are incubated for at least 7.5h in the dark in moisture-saturated atmosphere at 10-27°C. Exposure to light suppresses spore formation but allows sporophores to emerge from stomata. Incandescent or CW fluorescent light of 3.5 or 6 µmoles.m^2^.s^-1^ respectively, caused 100% inhibition of spore formation on lower leaf surface even when only the upper leaf surface was exposed to light. The inhibitory effect of light failed to translocate from an illuminated part of a leaf to a shaded part of the same leaf. Inhibition of sporulation by light was temperature-dependent. Light was fully inhibitory at 15-27°C but not at 10°C, suggesting that enzyme(s) activity and/or photoreceptor protein re-arrangement induced by light occur at ≥15°C. DCMU or paraquat could not abolish light inhibition, indicating that photosystem I and photosystem II are not involved. Narrow band led illumination showed that red light (λmax 625 nm) was most inhibitory and blue light (λmax 440 nm) was least inhibitory, suggesting that inhibition in *P. belbahrii*, unlike other oomycetes, operates via a red light photoreceptor. Nocturnal illumination of basil in the field (4-10 µmoles.m^2^.s^-1^ from 7pm to 7am) suppressed sporulation of *P. belbahrii* and reduced epidemics of downy mildew, thus reducing the need for fungicide applications. This is the first report on red light inhibition of sporulation in oomycetes and on the practical application of light for disease control in the field.

## Introduction

Downy mildew, caused by the biotrophic oomycete *Peronospora belbahrii* Thines [1,2], has recently become a major disease of sweet basil (*Ocimum basilicum* L) in many countries [3-11].

It was first observed in Northern Israel in November 2011. Within a month, the disease occurred simultaneously near the southwest and southeast borders of Israel, 250 km from the initial disease outbreak. By the summer of 2012, the disease had appeared throughout the country, causing major economic damage [12]. Within one year, mefenoxam became ineffective in controlling the disease due to the appearance of mefenoxam-resistant isolates of *P. belbahrii* [12].

Extensive research is currently underway around the world to identify fungicides effective for this disease. Gilardi et al [13] in Italy reported that, under glasshouse conditions, the best control was achieved with metalaxyl-M+copper hydroxide, a mineral fertilizer ‘Alexin’, mandipropanid, and azoxystrobin. Mersha et al [14] reported that acibenzolar-S-methyl (ASM, Actigard) and DL-3-aminobutyric acid (BABA) were effective in controlling the disease under greenhouse conditions in Florida. In Israel, two field experiments showed that the best performing fungicides, against a mefenoxam-sensitive isolate, are (in order) mefenoxam, mandipropamid, dimethomormph, and azoxystrobin (Cohen, Ben-Naim and Vaknin, unpublished data). 

Because of the strict regulations imposed by the Ministry of Agriculture on applications of chemicals to basil for the control of downy mildew, we looked for alternative methods to combat this disease. One such method is illumination during the dewy, dark sporulation period at night. The inhibitory effect of light on sporulation of downy mildew pathogens has been known for a long time[15]. Basil crops in Israel are grown under cover (plastic sheets or plastic nets), a fact that greatly enhances the feasibility of using light as a control measure. 

The objectives of the present study were to learn the effects of light on sporulation of *P. belbahrii* in growth chambers and then, to implement the results to disease control under commercial production conditions.

## Materials and Methods

### Plants

The sweet basil cultivar Peri (Volcani Center for Agricultural Research, Newe Ya’ar, Israel) was used in all experiments. For whole plant assays, plants were grown in 0.5L pots filled with peat:vermiculite (1:1, v/v) in the greenhouse (night/day temperature 18°C/32°C) and used for experiments at the 10-14 leaf stage. For detached leaf assays, plants were grown in the greenhouse in 1L pots filled with peat:compost (1:1, v/v) and inoculated at the 20-leaf stage. After inoculation plants were incubated at 24°C under constant illumination (CW fluorescent of 60 µmole.m^2^.s^-1^). Leaves having chlorotic lesions were harvested, placed on moist filter paper in plastic dishes, and used for sporulation assays (see below).

### Pathogen and inoculation

The mefenoxam-resistant isolate ‘Rehov-1’ of *P. belbahrii* was used in all experiments [12]. The isolate was maintained by repeated inoculation of whole plants in growth chambers at 20°C. Plants were inoculated by spraying a conidial suspension (5000 conidia per ml) on their upper leaf surface until run off. Inoculated plants were kept in a dew chamber (18°C) in the dark overnight and then transferred to a growth chamber at 24°C with continuous illumination (CW fluorescent light, 60 µmole.m^2^.s^-1^). 

### Sporulation assays

Infected leaves were detached from inoculated plants at 9-11 days post inoculation (dpi), placed on wet filter paper in 14cm Petri dishes or 20×20×2 cm dishes, and incubated in the dark or under light conditions at 20°C (unless specified otherwise) for 20 hours (unless specified otherwise). Sporulation intensity on individual leaves (n=6-8) was visually assessed using the following scale: 0= no sporulation; 0.5 = sparse sporulation; 1 = weak sporulation; 2 = moderate sporulation; 3 = heavy sporulation. The number of spores produced was counted in 1cm diameter leaf discs with aid of a cytometer. In some experiments with whole plants the number of leaves showing sporulation out of the total number of leaves in a plant were recorded and expressed as % sporulating leaves. 

### Microscopy

One-cm diameter discs were removed from each leaf, placed in 1 ml 50% ethanol and boiled for 5 minutes. Discs were removed, placed on a glass slide, lower surface upward, 100 µl 0.01% calcofluor in water was added to each disc, covered with a cover slip and examined with a Olympus-A70 microscope (Olympus, Japan) or Oxioimager-Z1 (Zeiss, Germany) epifluorescent microscope described previously [16]. A similar approach was used with whole infected plants except that plants were placed in transparent plastic bags to induce moisture-saturated atmosphere. 

### Light sources

In growth chambers, light was supplied by 20W Cool White (CW) or 40W CW fluorescent tubes, or 40W incandescent bulbs (Osram Sylvania IN, USA). To achieve color light we used the light-emitting diode Epro Lumen Aqua Led Lighting device model EPROA24S072W, 3W×24 pcs (Prodisc Technology Inc.Taipei, Taiwan). The emission spectra of the color led lights were measured with HR4000 spectrometer (Ocean Optics, Dundin, FL, USA). Light intensity was measured with Li-189 quantum sensor (Li-Cor, Lincoln, Nebraska USA). In the field, light was supplied with 20W Day Light (6400K) fluorescent bulbs (Leelite, China), 1 bulb per meter row. Each bulb was equipped with a white metal reflector (30 cm diameter). 

### Inhibitors of PSI and PSII

Intact infected plants were sprayed on upper leaf surface with various concentrations of the PSI photosynthesis inhibitor paraquat dichloride (methyl viologen dichloride, Syngenta-Makhteshim, Beer Sheba, Israel) or the PSII photosynthesis inhibitor DCMU ((3-(3,4-dichlorophenyl)-1,1-dimethylurea), Sigma)). Leaves were detached at 6h after spray and incubated for 20h at 20°C in the dark or under CW light of 45 µmole.m^2^.s^-1^. Sporulation and toxicity were visually estimated after 20h of incubation at 100% RH.

### Field experiments

The effect of light on sporulation of *P. belbahrii* was tested in the field during June-July 2013. The trials were conducted on Campus on private land belonging to Bar-Ilan University, Ramat Gan, Israel. The Dean of the Faculty of Life Sciences approved the trial. No protected animals or plants occur in this land. Three experiments were conducted in a net-house (6×45 meters), covered with a 50 mesh white plastic net equipped with 78 polystyrene containers (1.2×0.6×0.2 meter each, filled with about 120 liter of peat: vermiculite, 1:1 v/v). Containers were arranged in 3 rows, spaced 1 meter apart, 26 containers in a row. In an unrelated study, basil plants were planted in the right and the middle rows on mid April 2013 and artificially inoculated with *P. belbahrii* (isolate Rehov-1) on mid-May. These two rows served as an inoculum source for the experiment described below. 

The first experiment was performed on June 2, 2013, when10-leaf plants were planted in the left row, 10 clusters of 2-3 plant/cluster, per container. The western half of the row (13 containers) was illuminated daily from 19:00 pm until 07:00 am with 20W fluorescent bulbs (energy saving), 1 bulb per meter row. Bulbs, each equipped with a white metal cup reflector, were hung so as to provide light of 10 µmoles.m^-2^.s^-1^ at the plant apex level. The plants were illuminated starting on the day of planting. Plants in the left row started to show symptoms of downy mildew at 11 days after planting. They became infected by the spores of *P.belbahrii* dispersed from the neighboring two rows. Disease records were taken periodically by counting the number of healthy and infected leaves in each container. Periodically, leaves were detached and examined microscopically for spores.

The second experiment was performed on June 18, 2013. Ten-leaf plants were planted (10 plant clusters per container) in the middle and right row (which were evacuated a week earlier). Plants in these two rows were illuminated with 8 and 4 µmoles.m^-2^.s^-1^ in the manner described above starting on the day of planting. The infected plants in the left row served as inoculum source. The left row was evacuated and new, healthy10-leaf plants were planted on July 1, 2013 for the third experiment. Plants were illuminated with light intensity of 10 µmoles.m^-2^.s^-1^. Spores donated by the infected plants growing in the middle and right rows served as a source of inoculum for the left row.

### Statistics

Growth chamber studies were repeated three times or more and net-house experiments were done three times. One way analyses of variance were employed with JMP software and means were separated by using Turkey-Kramer HSD analysis with α=0.05. Means which were not connected with the same capital letter were significantly different.

## Results

### Time course of sporulation

**Figure 1:**
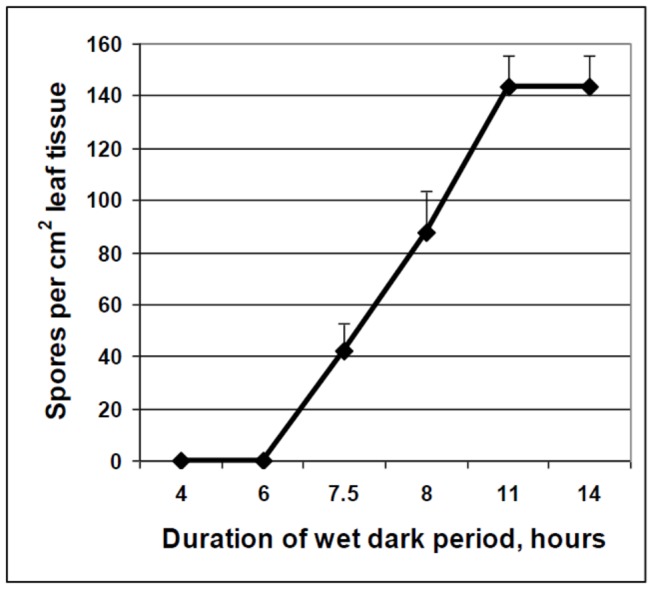
Sporulation of *Peronospora belbahrii* on intact basil plants as a function of time. Four-leaf plants were inoculated with isolate Rehov 1 and at 9 dpi were placed at in a dew chamber at 18°C in the dark. At the indicated time intervals, five plants were removed and one 11 mm diameter leaf disc was taken from leaf 3 and one from leaf 4 of each plant, placed in 50% ethanol solution and the number of spores counted.

**Figure 2:**
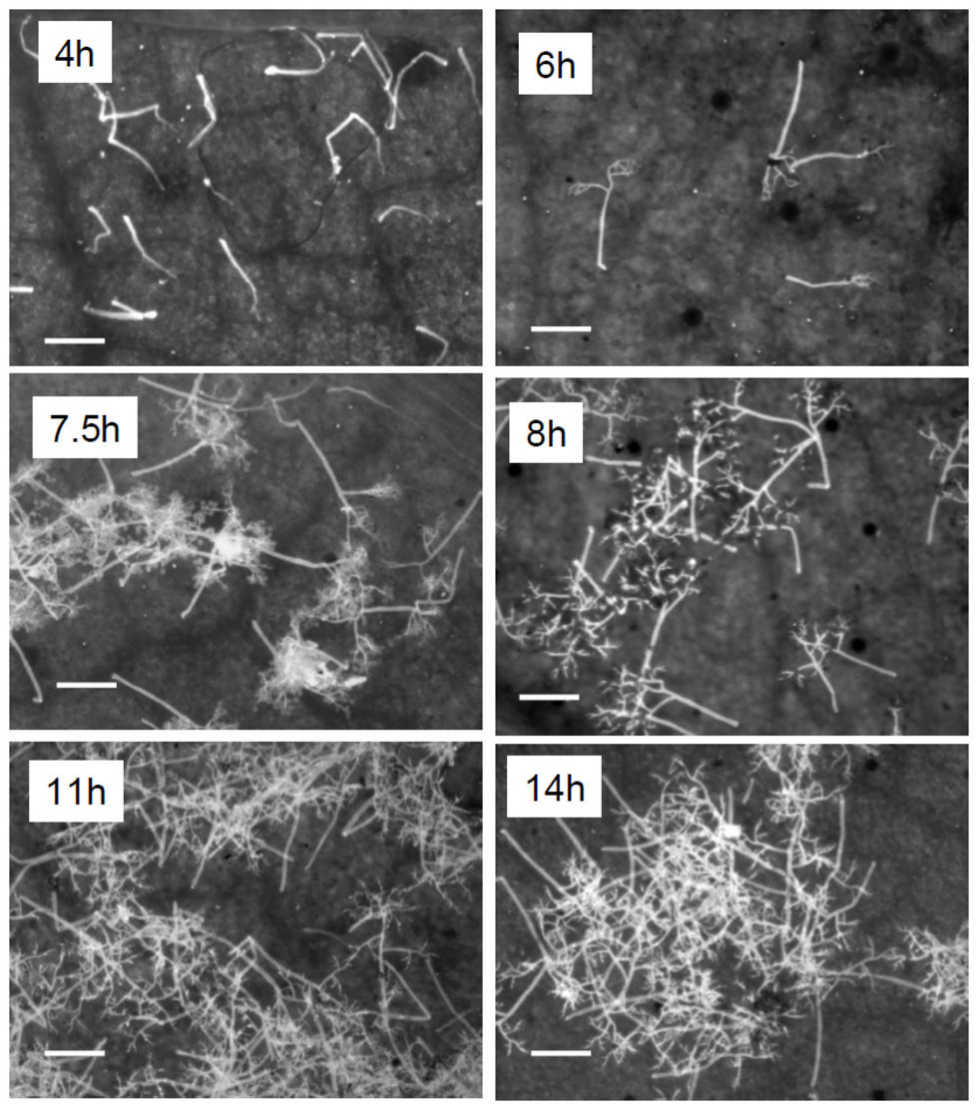
Microscopy of sporulation of *Peronospora belbahrii* on intact basil plants in a dew chamber at 18°C in the dark as a function of time (see Figure 1 for details). Leaf discs were mounted on a glass slide, treated with 0.01% calcofluor and examined with an Olympus-A70 epifluorescent microscope. Bar= 200 µm .

Emergence of sporophores from stomata of infected leaves started at 4 hours after placing theleaves in a humid atmosphere (100% RH) in darkness. Sporophores showed branching at 6h. Spores developed on sporophores at 7.5 hours and reached their maximal number (about 140×10^3^/cm^2^) at 11h (Figures 1 and 2). 

### Effect of light on sporulation

**Figure 3:**
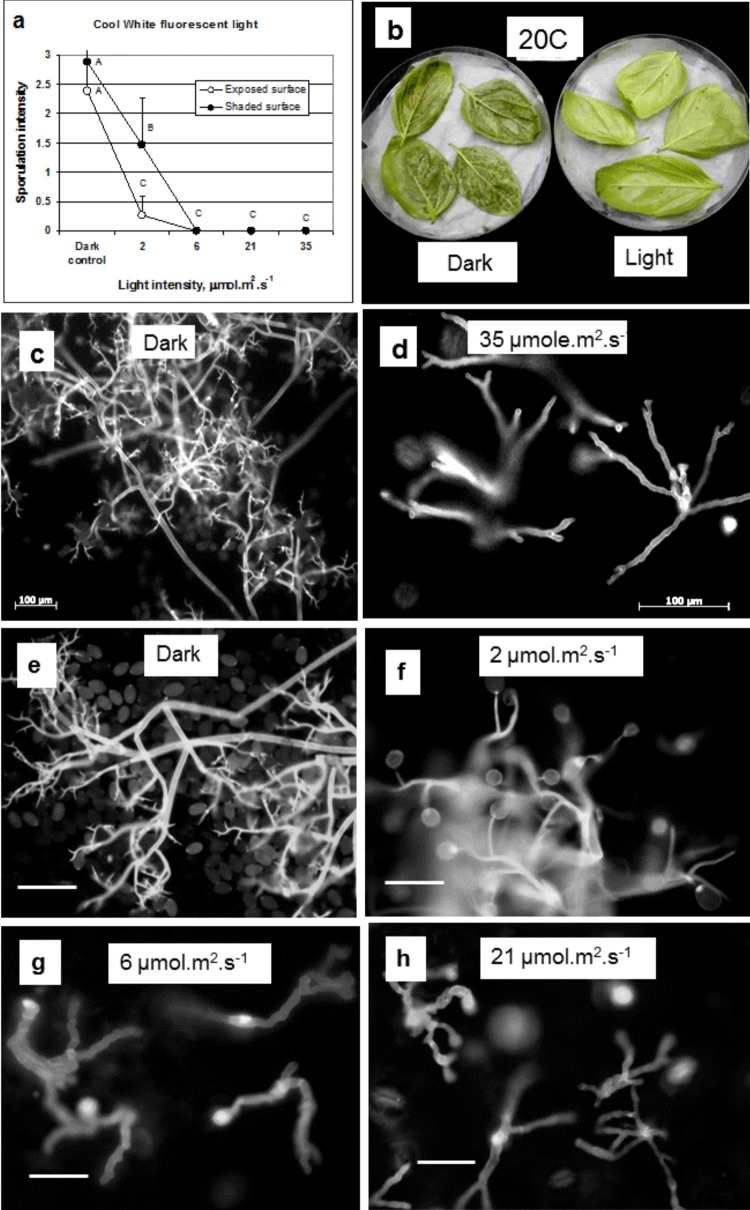
The inhibitory effect of light on sporulation of *Peronospora belbahrii* on detached basil leaves. Infected basil leaves were laid on a moist filter paper inside 14 cm Petri dishes, with their lower or upper leaf surface facing upwards, and incubated at 20°C for 20h in the dark or under CW fluorescent light of 2, 6, 21, or 35 µmol.m^2^.s^-1^. Images were captured with Oxioimager-Z1 (**c**-**d**) or Olympus A70 (**e**-**h**) epifluorescent microscopes. (**a**) Sporulation intensity on the lower leaf surface. Profuse sporulation occurred in the dark while no sporulation occurred under light, except at 2 µmol.m^2^.s^-1^. (**b**) sporulation on infected basil leaves incubated in the dark but not on leaves incubated under CW fluorescent light of 21 µmol.m^2^.s^-1^. (**c**-**h**) Fluorescent micrographs showing heavy sporulation in the dark (**c**,**e**) compared to sporophores bearing no spores under light of 6, 21, or 35 µmol.m^2^.s^-1^ (d.g,h). Note the formation of spores at 2 µmol.m^2^.s^-1^ (**f**).

Sporulation on the lower leaf surface of detached leaves was strongly suppressed by CW light at intensity of 6, 21, or 35 µmole.m^2^.s^-1^, regardless of whether the lower or upper surface of leaves faced the light (Figure 3a-h). At the lowest light intensity of 2 µmole.m^2^.s^-1^, inhibition of sporulation was partial, more so when the upper surface was exposed to light (Figure 3a,f). At ≥6 µmole.m^2^.s^-1^, sporulation was totally inhibited (Figure 3a,b,d,g,h). Fluorescent microscopy revealed that while dichotomously branched sporophores with abundant spores were formed in the dark (Figure 3c,e), abnormally-branched sporophores, with no spores, were formed under light conditions (Figure 3d,g,h).

**Figure 4:**
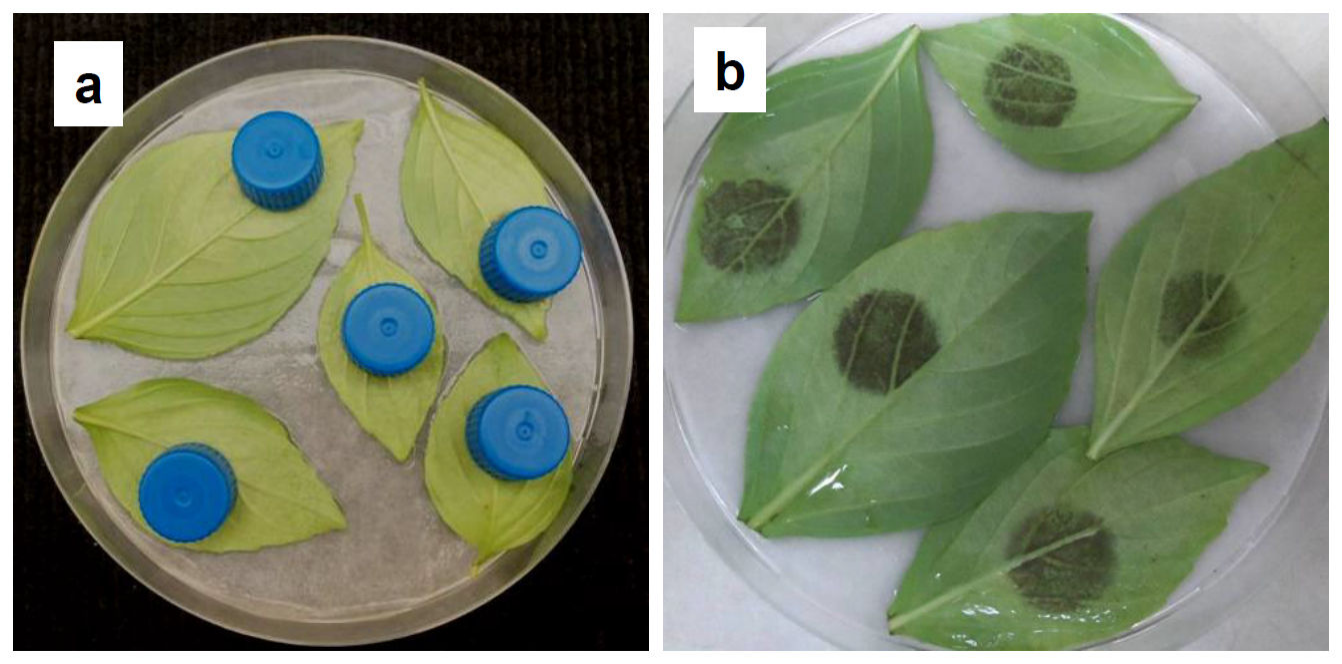
The local inhibitory effect of light on sporulation of *Peronospora belbahrii* on detached basil leaves. Opaque plastic caps of 21 mm diam. were placed on infected basil leaves laid on moist filter paper inside 14 cm Petri dishes. Plates were incubated at 20°C under CW fluorescent light of 35 µmol.m^2^.s^-1^. Caps were removed after 20h. Sporulation occurred only underneath the caps.

The inhibitory effect of light was restricted to the illuminated area of the leaf. Profuse sporulation occurred in the round areas of the leaf that were darkened by test tube covers, but not around them (Figure 4) suggesting that no soluble inhibitor has translocated from the illuminated zone to the dark zone. 

### The interaction effect of light and temperature on sporulation

**Figure 5:**
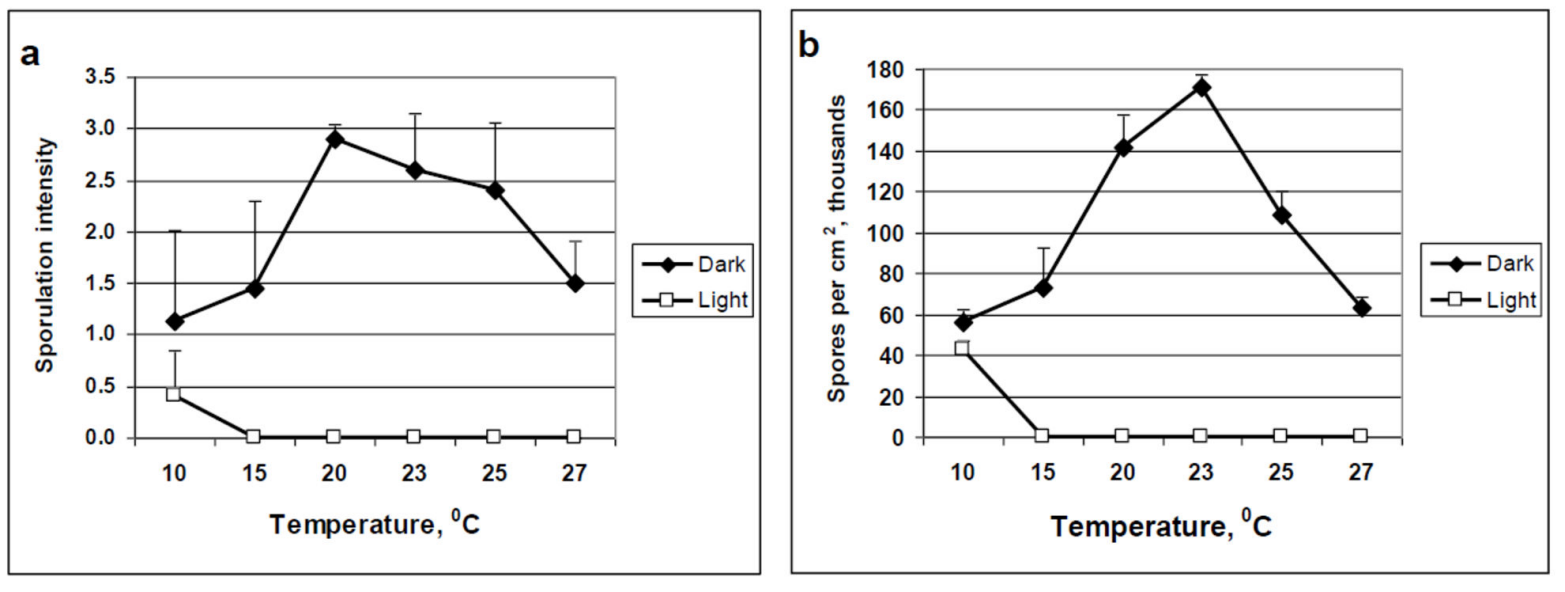
The interacting effect of light and temperature on sporulation of *Peronospora belbahrii* on detached basil leaves. Infected basil leaves were laid on a moist filter paper inside 14 cm Petri dishes, and incubated at 6 temperatures from 10°C to 27°C for 20h in the dark or under CW fluorescent light of 21 µmol.m^2^.s^-1^. (**a**) Visual estimation of sporulation intensity (**b**) Number of spores per cm^2^ (one 11 mm diameter leaf disc was taken from each leaf, placed in 50% ethanol and the number of mature spores counted) Sporulation values under light are significantly lower compared to values in darkness, except at 10°C.

**Figure 6:**
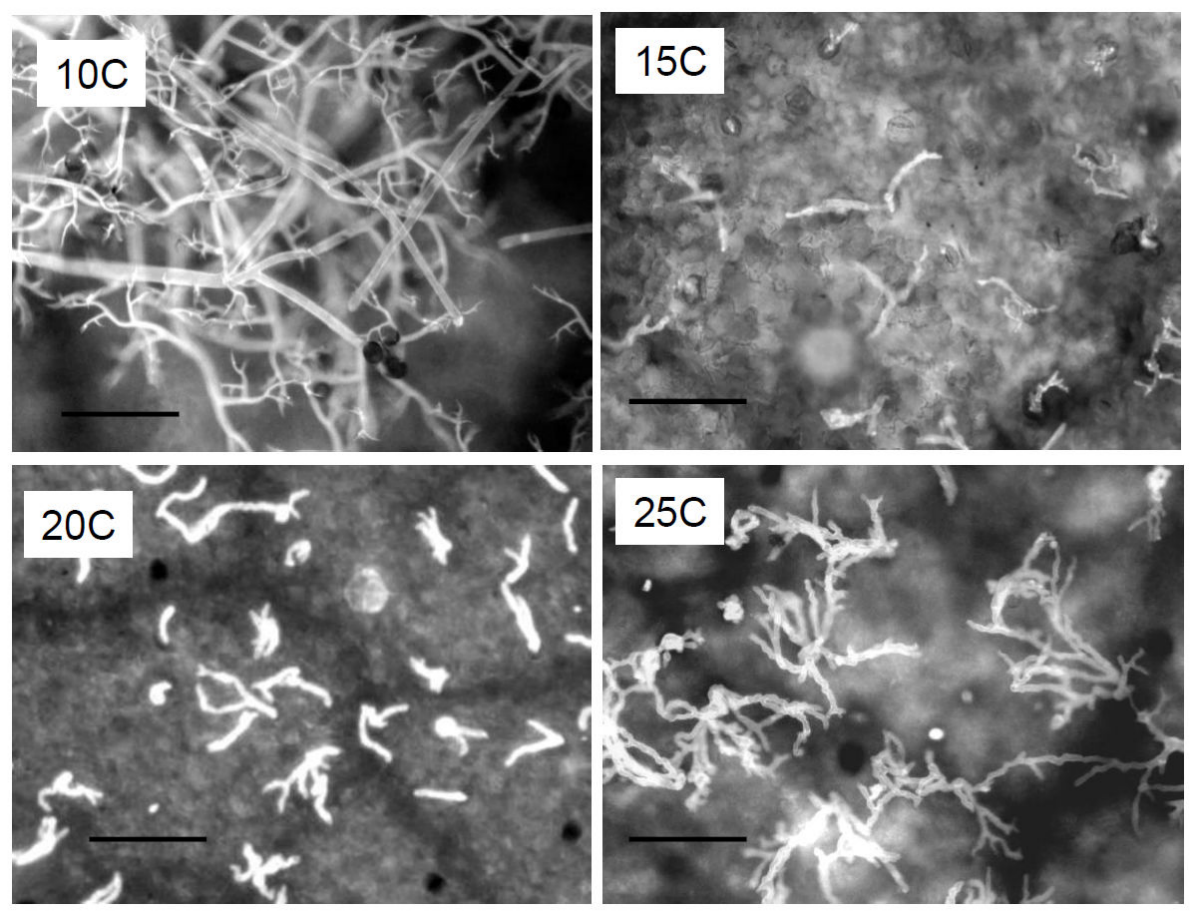
Fluorescent micrographs showing the influence of CW fluorescent light of 21 µmol.m^2^.s^-1^ on sporulation of *Peronospora belbahrii* at various temperatures. At 10°C normal sporophores and spores were formed, whereas abnormal sporophores with no spores were formed at 15, 20 and 25°C. At 25°C sporophores were abundant.

Our data support previous research conducted with other Peronosporales showing that light inhibits sporulation at ≥15°C. The data provided here corroborate with those results. Light was strongly inhibitory to sporulation of *P. belbahrii* in leaves incubated at 15-27°C but not in leaves incubated at 10°C (Figure 5). Epifluorecent micrographs showed branched sporophores bearing spores at 10°C, but only sporophores at 15-25°C (Figure 6).

### The effect of light quality on sporulation

**Figure 7:**
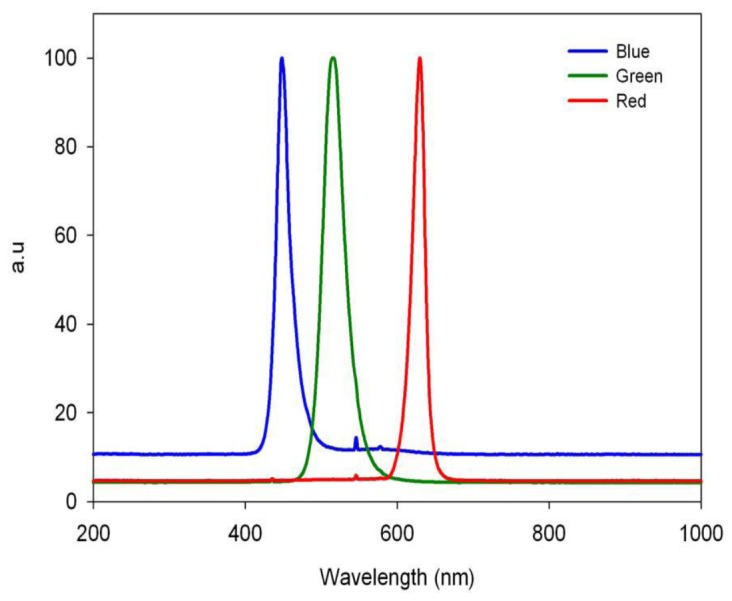
Emission spectrum of led light sources used in this study (Epro Lumen Aqua led Lighting model EPROA24S072W, 3W×24pcs (Prodisc Technology Inc.Taipei, Taiwan). Peak wavelengths are: blue- 440 nm; green- 500 nm; red- 625 nm.

**Figure 8:**
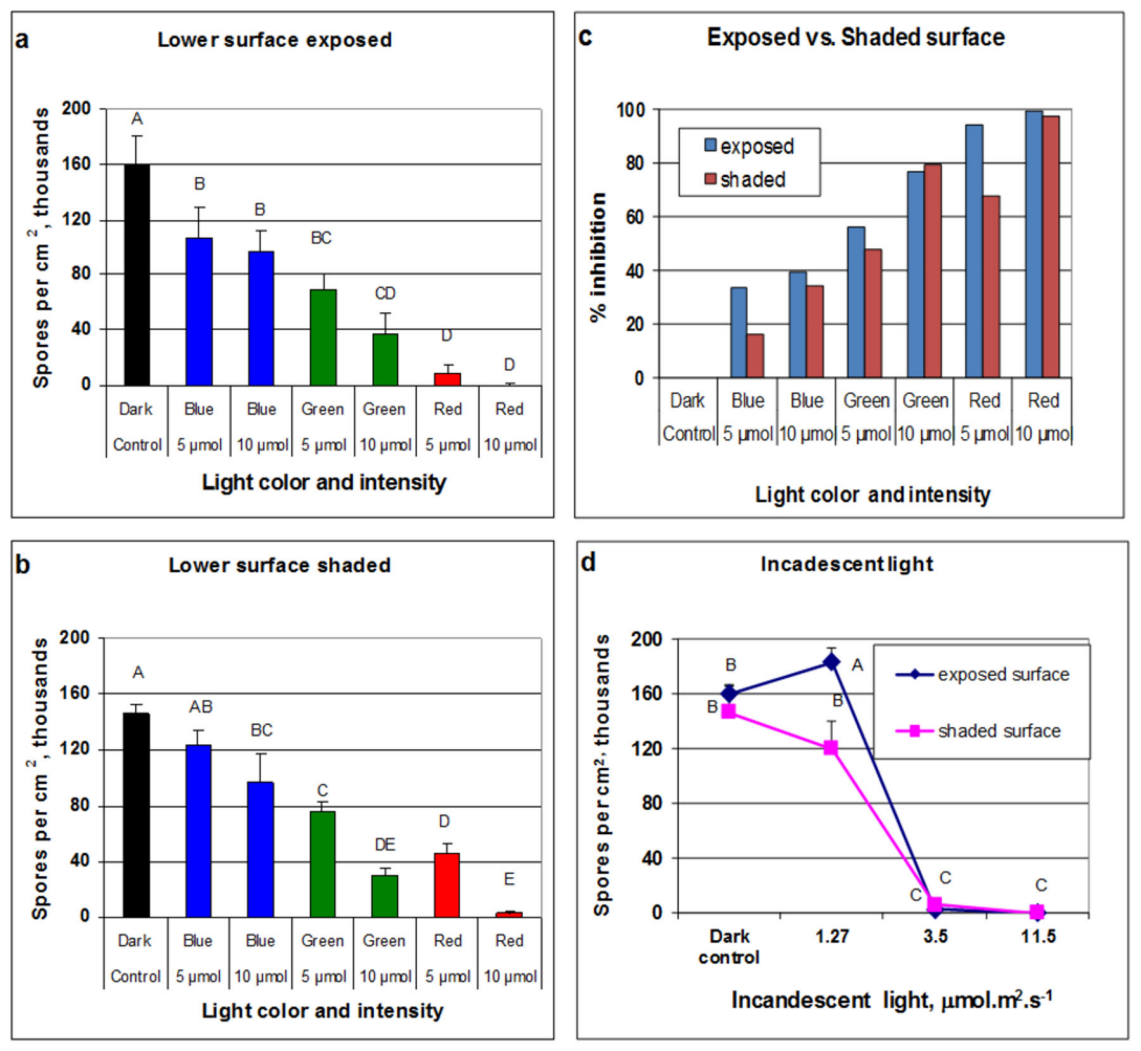
Effect of light quality and intensity on sporulation of *Peronospora belbahrii* on detached basil leaves. Infected basil leaves were laid on a moist filter paper inside 20×20 cm Nunk dishes, 6 leaves with their lower and 6 leaves with their upper leaf surface facing upwards. Dishes were incubated at 20°C under blue, green, or red led lights of 5 or 10 µmol.m^2^.s^-1^, or under incandescent light of 1.27, 3.5, or 11.5µmol.m^2^.s^-1^. After 20h of incubation, 1cm diam. leaf disc was removed from each leaf and number of spore counted. (**a**) sporulation on lower leaf surface in leaves whose lower surface was exposed to led lights. (**b**) sporulation on lower leaf surface in leaves whose upper surface was exposed to led lights (**c**) % inhibition of sporulation relative to dark control as calculated from (**a**) and (**b**). (**d**) Inhibition of sporulation by incandescent light. (For the effect of CW light see Figure 5).

Led light sources with narrow emission spectra were used (Figure 7). Leaves were exposed to light under moist conditions at 20°C with their lower or upper surface faced to light, and sporulation was quantified at 20h. Results are presented in Figure 8. Red light was most inhibitory, whereas blue light was least inhibitory, regardless of the leaf surface facing light (Figure 8a-c). Illumination with 10 µmole.m^2^.s^-1^ was more inhibitory than illumination with 5 µmole.m^2^.s^-1^. Percentage inhibition, relative to dark control, induced by blue, green or red light of 10µmole.m^2^.s^-1^ applied to the lower surface was 39.6, 77.1 and 99.7%,respectively (Figure 8c). Incandescent light, which includes red and near infrared light, was almost fully inhibitory at 3.5 µmole.m^2^.s^-1^, regardless of the leaf surface exposed to light (Figure 8d).

### Inhibitors of PSI and PSII

**Figure 9:**
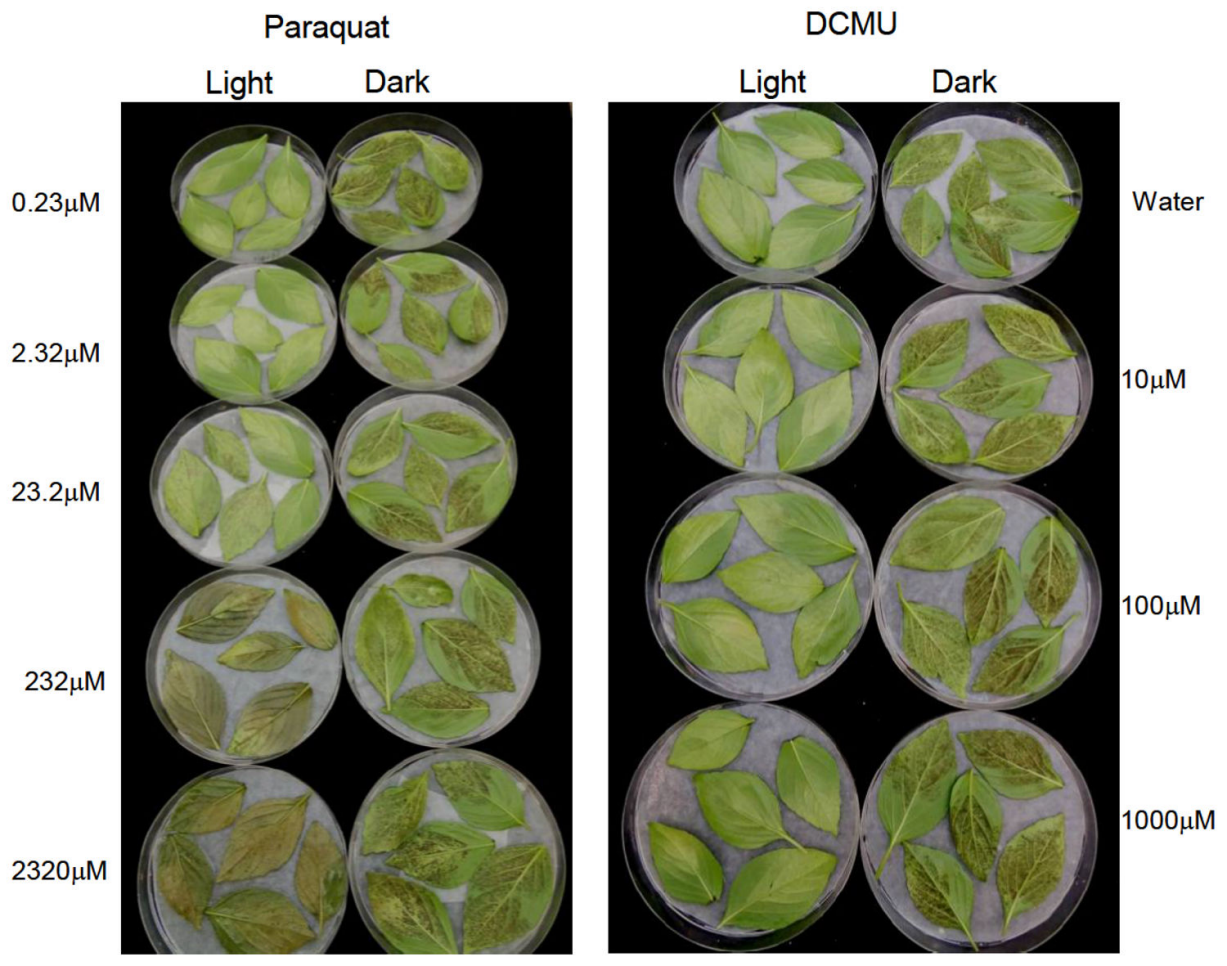
Light suppresses sporulation of *Peronospora belbahrii* in basil leaves treated with the photosynthesis inhibitors paraquat or DCMU. Infected plants were sprayed with various concentrations of paraquat (0.23-2320 µM) or DCMU (10-1000 µM). At 6h after spray leaves were detached, placed on moist filter paper in 14cm Petri dishes and incubated at 20°C in the dark or under CW fluorescent light of 45 µmol.m^2^.s^-1^for 20h. Light prevented sporulation in all leaves, but allowed sporophore emergence, regardless of the treatment. Paraquat at the two highest concentrations was toxic to leaves exposed to light but not to those incubated in darkness.

Results presented in Figure 9 show that neither paraquat nor DCMU could abolish the inhibitory effect of light on sporulation. In the dark, profuse sporulation occurred in leaves treated with DCMU of 0, 10,100 and 1000 µM, and no toxicity to leaves was observed. In the light, sporophores emerged from all leaves regardless of DCMU concentration. Leaves treated with paraquat of 0.23, 2.3, 23, 230, or 2300 µM showed profuse sporulation in the dark, with no toxicity seen. Leaves incubated under light showed sporophores with paraquat of 0.23, 2.3 and 23 µM but strong toxicity (advanced killing) at 232 and 2320 µM. Paraquat was herbicidal (producing ROS) under light conditions only [17].

### Field experiments.

**Figure 10:**
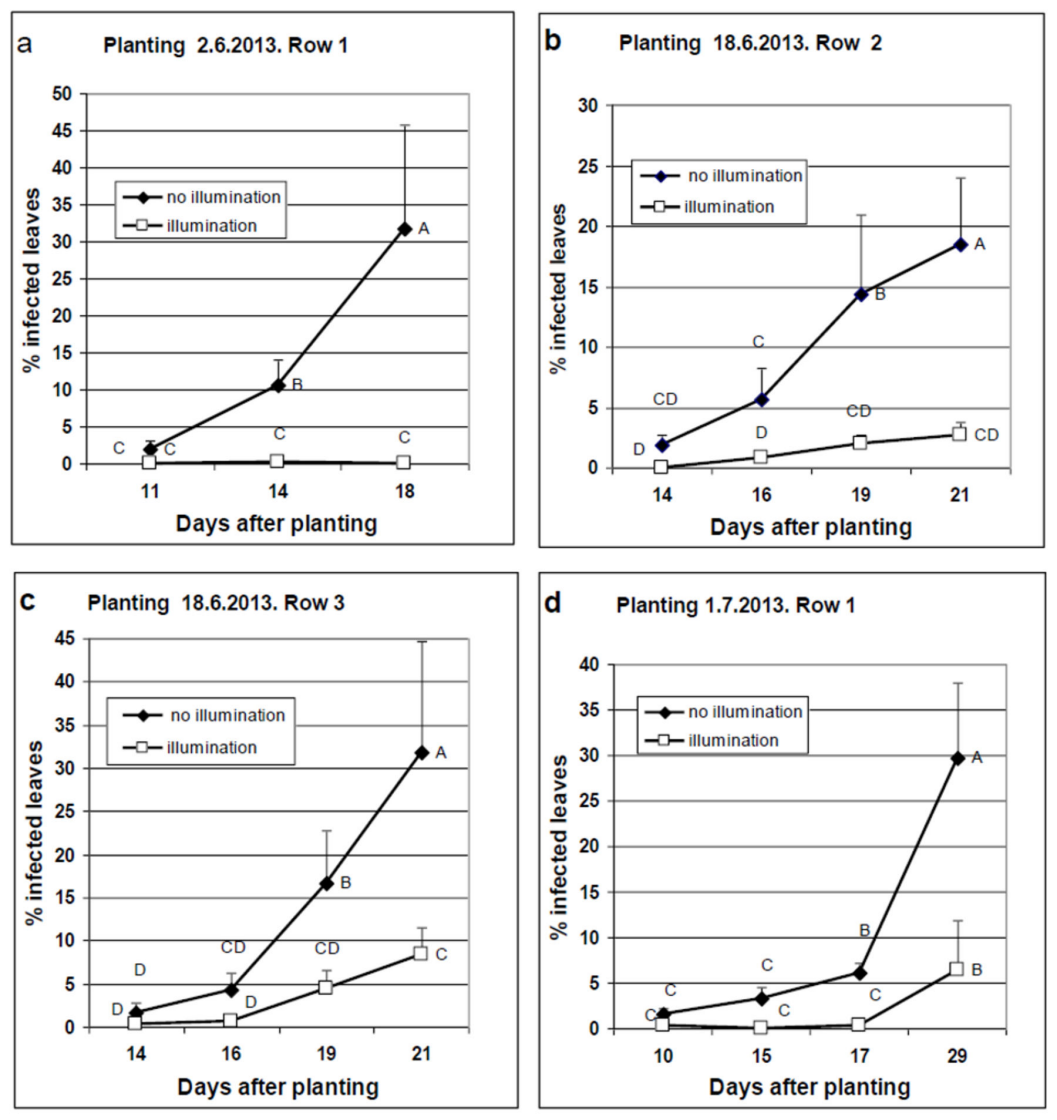
Results from net-house experiments documanting the effect of illumination during the night on epidemics of downy mildew in basil crops. Ten leaf basil plants were planted in three rows of 26 containers each (see Materials and Methods) in a net house located on campus. The 13 eastern containers were illuminated daily from 19:00 pm to 07:00 am, while the 13 western containers in each row remained un-illuminated, serving as controls. Illumination was supplied by 20W “Day Light” fluorescent bulbs. (**a**), (**b**)**, (c)** and (**d**), plants were planted on 2.6.2013, 18.6.2013, 18.6.2013, and 1.7.2013, respectively. Bulbs were hung so that the light intensity reaching the apices of the plants at day of planting was 10, 8, 4, and 10 µmol.m^2^.s^-1^, respectively. Light intensity changed during the epidemic due to plants growth. Plants of the first experiment (2.6.2013) became infected from spores dispersed from the adjacent two rows which were artificially inoculated 3 weeks earlier. Plants of the first experiment served as a source of inoculum for plants of the second experiment (18.6.2013), etc. At the indicated time intervals after planting, the number of symptomatic and healthy leaves in each container was counted and the proportion of infected leaves was calculated. Bars show the standard deviation of the means (n=13), and different letters indicated significant differences between means (Tukey-Kramer HSD analysis, α=0.05).

In three experiments, nocturnal illumination significantly suppressed disease development under field conditions (Figure 10). In the first experiment, in which plants were exposed to quanta of 10 µmol.m^2^.s^-1^, no disease was recorded in illuminated plants until 18 days after planting, whereas a mean of 32% infected leaves was counted in control un-illuminated plants Figure 10a). In the second experiment, illuminated plants were exposed to quanta of either 8 µmol.m^2^.s^-1^ (Row 2, Figure 10b) or 4 µmol.m^2^.s^-1^ (Figure 10c, Row 3). Illumination significantly suppressed disease development in both rows. However, % protection provided by 8 µmol.m^2^.s^-1^ (Row 2) ranged between 85-97%, whereas % protection provided by 4 µmol.m^2^.s^-1^ ranged between 74-83%. In the third experiment, illuminated plants were exposed to quanta of 10 µmol.m^2^.s^-1^. Percent protection was 95.6% and 78.6% at 17 and at 29 days after planting, respectively (Figure 10d). In all field experiments, illumination suppressed spore formation but not emergence of sporophores from stomata. Protection level was less pronounced as disease progressed due to shading caused by the newly developed leaves and increased concentration of dispersed spores in the air.

**Figure 11:**
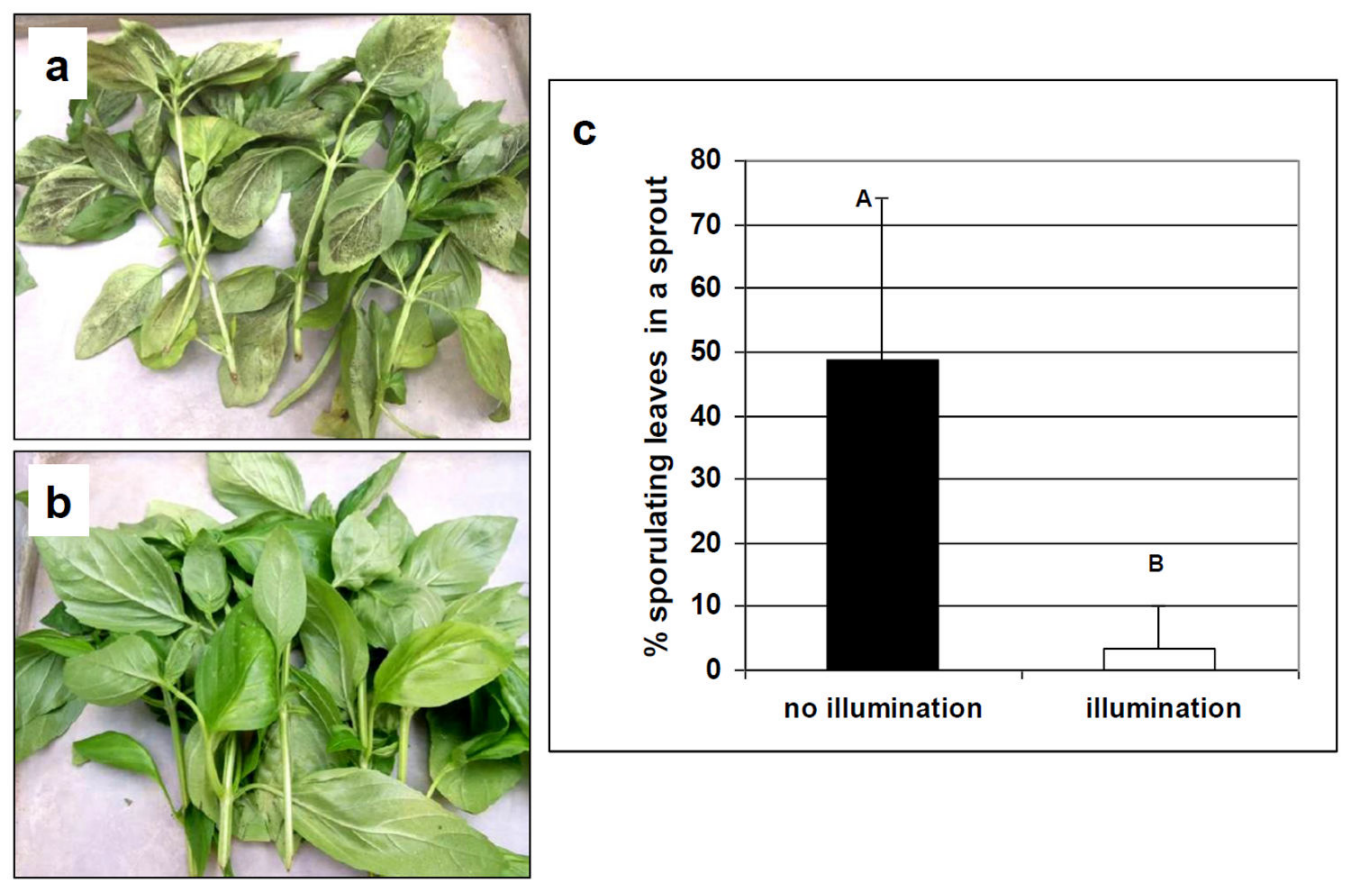
Illumination during the night suppressed disease development in newly-developed basil sprouts in the field. Plants in row 3 (80 cm tall, planted on 18.6.2013) were cut down at 21 days after planting to a height of 20 cm. The cut branches were discarded. A week later, 5 sprouts (each having 8-12 leaves) were collected from each container and the number of sporulating leaves was counted. (**a**) Sporulation on sprouts collected from control un-illuminated plants. (**b**) Sporulation on sprouts collected from illuminated plants. (**c**) Proportion of sporulating leaves in a sprout (n=45).

A strong protective effect of nocturnal illumination was seen after harvest. Plants in Row 3 were cut down at the end of the second experiment to a height of 20 cm. The sprouts that developed after a week were mostly free of the disease when they developed under nocturnal illumination, whereas sprouts that developed on control un-illuminated plants were heavily infected (Figure 11a-c).

To examine the effect of illumination during the wet inoculation period on infection, intact 10-leaf basil plants were placed after spray inoculation in moist chambers at 20°C for 20h in the dark or under CW light of 0, 5, 15, 30, and 45 µmol.m^2^.s^-1^. At 10 dpi plants were placed in a dew chamber (18°C, darkness) for 20h to induce sporulation. All plants showed heavy sporulation, regardless of the light regime during infection. This suggests that disease suppression induced by light in the field has not resulted from light inhibition of infection. 

## Discussion

The asexual sporulation of *Peronospora belbahrii*, the causal agent of basil downy mildew, is strongly affected, as are other biotrophic foliar plant pathogens of the Oomycetes, by host and environmental factors. Major host factors are lesion developmental state and pool size of photosynthetic metabolites. The major environmental factors are air humidity, temperature and light.

Asexual sporulation of downy mildew causal oomycetes is a light-sensitive process. On the one hand, light stimulates sporulation via photosynthesis of the host by making hexoses available for spore formation, and on the other hand, light suppresses sporulation at its terminal phase as it can progress in the dark only [15].

Spores (sporangia or conidia) are formed on the termini of branched sporophores which emerge from leaf stomata. This process occurs in moisture-saturated atmosphere in the dark and requires 8-12 hours to complete at an appropriate temperature. Sporulation starts when pathogen colonization has reached a certain level of density (biomass) in the mesophyll and terminates when the leaf becomes necrotic. The intensity (abundance) of sporulation is strongly dependent on the preceding photosynthetic conditions. The greater the accumulation of carbohydrates in the infected leaf the greater number of spores will be produced in darkness. Longer photoperiods or continuous preceding light enhance sporulation in the following dark wet period. The carbohydrates accumulating during the light period are hydrolyzed to hexoses during the first half of the night and used by the pathogen to build its sporophore and spore walls during the second part of the night. When light is provided during the second half of the night, no spores are formed. Blue light is more inhibitory to sporulation than red or green light [15].

*P. belbahrii* sporulates in chlorotic lesions whose age range between 5-15 days; enhanced lighting during lesion development increases subsequent sporulation in the dark (Cohen et al, *unpublished data*).

The process of sporulation in *P. belbahrii* is completed within about 11 hours from the onset of darkness in moisture-saturated atmosphere at 18°C. During the first 6 hours, hyaline sporophores emerge from stomata which gradually become dichotomously-branched, and during the subsequent 5 hours dark spores appear on the tips of the sporophore branchlets.

Light strongly inhibits the sporulation of *P. belbahrii* on basil leaves. Light inhibits spore formation but not the emergence of sporophores from stomata. Sporophores produced under light are abnormal and fail to form spores. This corroborates with previous findings with several other Peronosporales, including *Sclerospora sorghi* in maize [18], *Pseudoperonspora cubensis* on cucumber [19], *Peronospora tabacina* on tobacco [20], *Phytophthora infestans* on potato [21] and *Plasmopara viticola* on grapes [22]. In all cases, spore production, but not sporophore formation, was inhibited by light. Normally, one straight sporophore emerged from a stoma under light conditions. In many occasions, however, it ramificated at the stoma opening level, producing 2-4 sporophore arms whose shape greatly varied, from a rosette to long curled shape. Light intensity, light quality, and temperature during the wet sporulation period dictated the suppressing effects on spore formation. 

The higher the intensity the stronger the inhibitory effect of light on spore production. An inhibitory effect was already seen at low intensity of 2 µmol.m^2^.s^-1^ of CW fluorescent light. Full inhibition was achieved with ≥6 µmol.m^2^.s^-1^ regardless of the leaf surface exposed to light. This seems surprising, as our measurements show that an infected chlorotic basil leaf absorbs 89% of the CW light it is exposed to, resulting in 11% of the light quanta reaching its lower surface. When a leaf is exposed to 6 µmol.m^2^.s^-1^ its lower surface “sees” only ~0.6 µmol.m^2^.s^-1^. At this intensity no inhibition of sporulation should occur. This calculation suggests that the photoreceptors activated by light may reside inside the mesophyll, probably in the hyphae and not solely at the sporophore tip. 

The inhibitory effect of light is local. The leaf area exposed to light during the wet period showed no sporulation, while the adjacent area covered with a dark screen showed abundant sporulation. This indicates that no inhibitory factors translocate from the illuminated area into the darkened area. This result is different from the P. *tabacina* – tobacco system in which some inhibition was measured in the darkened area of an illuminated leaf [20]. The difference may be attributed to leaf size: the tobacco leaves used were 5 times larger than the basil leaves used here. This point warrants further investigation.

Narrow light spectra provided by led illumination showed that red light was significantly more inhibitory to spore formation compared to blue light. This finding is in contrast with previous research showing that blue light was more inhibitory than red light in suppressing sporulation of *P. cubensis* on cucumber leaves [19], *P. tabacina* on tobacco leaves [20], *P. infestans* on potato leaves [21], and *Plasmopara viticola* on grape leaves [22]. One possible explanation to this discrepancy is that *P. belbahrii* has a different photoreceptor, sensitive to red light.

It is not known whether oomycetes carry red light photoreceptors. True fungi contain genes for different putative photoreceptors, such as flavin-binding proteins, phytochromes, cryptochromes and opsins. These proteins use different chromophores (low-molecular-weight cofactors that enable a protein to absorb light of specific wavelengths) that allow them to detect different wavelengths. Thus, phytochromes have a bilin chromophore that absorbs red light, opsins use retinal to detect green light, and flavin-binding photoreceptors detect blue light [23-26]. Fungal phytochromes act as light-regulated histidine kinases. Hypothetical model of red light signaling in *Aspergillus nidulans* [24] shows the mechanism of signal transduction from the primary light absorption event of the chromophore to protein conformational changes leading to protein interactions and gene expression regulation [24]. After 30 min of illumination, *2*09 genes were up- regulated and 51 down-regulated. Some photo-inducible genes encode transcription factors and enzymes and others are involved in stress responses.

Gene expression was investigated in *P. infestans* during sporulation on tomato leaves [27]. Visible sporulation began in the middle of the fourth night after infection (94h). Sporulation continued until the following dawn, and resumed during the fifth night. RNA was then measured by qRT-PCR at 48 and 72h (pre-sporulation) and 96h (post-sporulation), using primers for 73 genes. All but two showed significant induction in the 96h sample as compared to the pre-sporulation time points. Which genes were involved in light suppression of sporulation was not investigated.

During evolution, downy mildew agents acquired the trait of sporulating at night, when dew occurs. A reasonable assumption would be that light serves as an information signal saying that external conditions are stressful (dry) and inappropriate for spore production. 

We show here that the photosynthetic machinery of the host is probably not responsible for the inhibitory effect of light on sporulation. One reason is that light quantity required to suppress sporulation is too low to activate significant photosynthesis in the host. Another reason is that in the presence photosynthesis inhibitors [17], sporulation under light was still prevented. Neither paraquat, inhibitor of PSI, nor DCMU, inhibitor of PSII, could abolish the inhibitory effect of light on spore formation. Both compounds did not prevent sporophore growth (except paraquat at 232 µM and 2320 µM), suggesting that that they bear no direct toxicity toward the pathogen. The fact that photosynthesis is not involved does not necessarily mean that the photoreceptor does not reside in the host cells. It would be, however, reasonable to assume that a postulated photoreceptor occurs in the pathogen. It may be harbored by the mycelia colonizing the leaf, the sporophores that emerge out, or both. 

The inhibitory effect of light on spore formation was temperature-dependent. Light prevented spore formation at 15-27°C but not at 10°C. This was found true with other downy mildews and true fungi as well [15] There are at least two possible explanations for this phenomenon: (i) light activates an enzyme buildup of anti-sporulation compounds in the irradiated tissue at high temperatures (15-27°C). (ii) the structural re-conformation of the photoreceptor which happens as a result of light excitation operates at high but not low temperatures. More research is needed to clarify this phenomenon.

Light and temperature also interact during sporophore formation under moist conditions. At 15°C, light allows the formation of 1 or 2 short sporophores per stoma; at 20°C, 2 or 3 longer sporophores are produced per stoma; and at 25°C, 3 or 4 long abnormally-branched sporophores are produced per stoma. It seems that a high temperature enhances sporophore growth as it does with mycelial growth inside the leaf tissue during colonization. 

While the inhibitory effect of light on sporulation of downy mildews has been known for many years, our study is the first to show that light can be used to control disease under field conditions. In a series of experiments we showed that nocturnal illumination of basil crops in the field, from 7 pm to 7 am, was strongly effective in suppressing the sporulation of *P. belbahrii* and as a result, reducing the proportion of infected leaves. Nocturnal illumination with Day Light fluorescent bulbs at an initial intensity of 4, 8, or 10 µmol.m^2^.s^-1^ reduced the number of sporulating leaves by 70, 85 and 100%, respectively, relative to non-illuminated plants. This high level of protection persisted for 2-3 weeks after planting/inoculation and was comparable to fungicidal treatments. Our growth chamber studies revealed that potted basil plants developed the same amount of symptoms and sporulation regardless of whether kept during the first day after inoculation under light or dark conditions. This strengthens our observation that inhibition of spore formation is the major reason for disease suppression in the field. 

 Farmers employ the practice of cutting low their basil plants after several harvests in order to renew their sprouting. The efficacy of nocturnal illumination after such cutting was very significant. The proportion of infected leaves in the newly developed sprouts was 10 times lower in illuminated as compared to non-illuminated plants. 

The data presented in this paper suggests that light can successfully be used to suppress downy mildew development in basil in the field. This is the first report showing the control of a downy mildew disease in the field by nocturnal illumination.
